# Cryo-EM: A new dawn in thyroid biology

**DOI:** 10.1016/j.mce.2021.111309

**Published:** 2021-07-01

**Authors:** Francesca Coscia, Ajda Taler-Verčič

**Affiliations:** a MRC Laboratory of Molecular Biology, Cambridge, CB2 0QH, UK; b Human Technopole, Via Cristina Belgioioso 171, 20157, Milano, Italy; c University of Ljubljana, Faculty of Medicine, Institute of Biochemistry and Molecular Genetics, Vrazov Trg 2, 1000, Ljubljana, Slovenia

**Keywords:** Thyroid hormones, Iodine, Cryo-EM, Electron microscopy, Structural biology, Thyroglobulin

## Abstract

The thyroid gland accumulates the rare dietary element iodine and incorporates it into iodinated thyroid hormones, utilising several tightly regulated reactions and molecular mechanisms. Thyroid hormones are essential in vertebrates and play a central role in many biological processes, such as development, thermogenesis and growth. The control of these functions is exerted through the binding of hormones to nuclear thyroid hormone receptors that rule the transcription of numerous metabolic genes. Over the last 50 years, thyroid biology has been studied extensively at the cellular and organismal levels, revealing its multiple clinical implications, yet, a complete molecular understanding is still lacking. This includes the atomic structures of crucial pathway components that would be needed to elucidate molecular mechanisms. Here we review the currently known protein structures involved in thyroid hormone synthesis, regulation, transport, and actions. We also highlight targets for future investigations that will significantly benefit from recent advances in macromolecular structure determination by electron cryo-microscopy (cryo-EM). As an example, we demonstrate how cryo-EM was crucial to obtain the structure of the large thyroid hormone precursor protein, thyroglobulin. We discuss modern cryo-EM compared to other structure determination methods and how an integrated structural and cell biological approach will help filling the molecular knowledge gap in our understanding of thyroid hormone metabolism. Together with clinical, cellular and high-throughput 'omics' studies, atomic structures of thyroid components will provide an important framework to map disease mutations and to interpret and predict thyroid phenotypes.

## Highlights

• Iodine cycle and thyroid hormones in vertebrates: a molecular view.
• 3D structures of proteins involved in thyroid hormone metabolism.
• Recent advances in cryo-EM.
• Thyroglobulin cryo-EM structure determination: a case study.
• Towards a complete mechanistic understanding of thyroid biology.

## 1 Iodine cycle and thyroid hormones in vertebrates: a molecular view

**Fig. 1:**
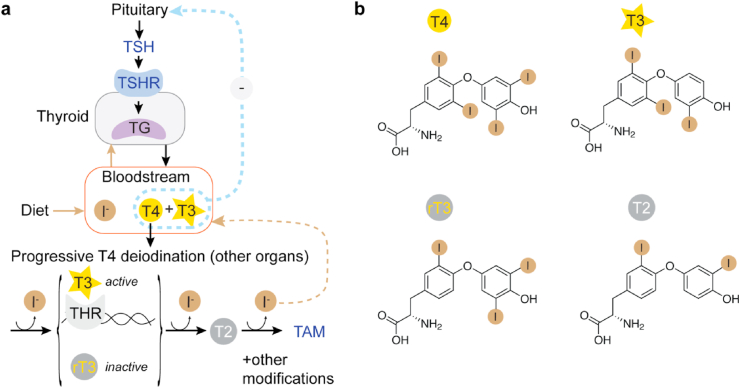
**Schematic overview of the thyroid hormone cycle.** a) Thyroid-pituitary axis and general thyroid hormone pathway in vertebrates. TSH: thyroid-stimulating hormone, TSHR: TSH receptor, TG: thyroglobulin, T4: 3,5,3′,5′-tetraiodo-L-thyronine, T3: 3,5,3′-triiodo-L-thyronine, THR: thyroid hormone receptors, rT3: 3,3′,5′-triiodo-L-thyronine, T2: 3,3′-diiodo-L-thyronine, TAM: thyronamine derivatives. b) Molecular structure of the thyroid hormones (TH): T4, T3, rT3 and T2.

Iodine has one of the largest atomic diameters present in biological systems, and it exists mainly in ionic forms such as iodide (I^−^) or iodates (IO_3_^−^). Evolutionarily, it has been hypothesised that iodine, together with other halogens (mainly chlorine and bromine), was first used by early planktonic organisms as an antioxidant agent to buffer oxygen species via peroxidase enzyme activity (Taurog, 1999; Venturi, 2011). Probably derived from this primordial function, several marine species (especially algae) then developed iodide accumulation systems and the ability to synthesise a variety of iodinated organic compounds (Küpper et al., 2008; Küpper and Carrano, 2019; La Barre et al., 2010). Tunicates were likely the first organisms where the iodinated compounds 3,5,3′,5′-tetraiodo-L-thyronine (thyroxine, T4) and 3,5,3′-triiodo-L-thyronine (T3) appeared. They have been then maintained throughout subsequent evolution and are still produced in present-day vertebrates as thyroid hormones (TH) (Crockford, 2009; Dembitsky, 2006; Holzer et al., 2017). Because of their organism-wide effects on metabolism and development, thyroid hormones (TH) are essential in all vertebrates, making them strictly dependent on iodine intake (Carvalho and Dupuy, 2017). Typically, iodine is found as a dietary compound in algae, iodine/sea salts and seafood. Throughout evolution and the transition to terrestrial environments with very little available iodine, accumulation systems for iodine and compartments have developed in primitive endostyles. This has culminated in the modern follicular thyroid gland system that is shared by humans and that allows efficient iodine storage and tight control of TH synthesis (De Felice and Di Lauro, 2011; Nilsson and Fagman, 2017). Initially, iodide is taken up by the thyroid gland where it undergoes compartmentalised oxidation and organification into TH (Fig. 1). Subsequently, TH undergo progressive de-iodination steps to produce compounds that are active in metabolism and also signalling. The resulting iodide is recycled into the bloodstream where it becomes available for further storage in the gland. A first de-iodination event can either activate or deactivate T4 by converting it into T3 or rT3 (3,3′,5′-triiodo-L-thyronine), respectively. In all vertebrate cells, DNA-binding thyroid hormone receptors (THR), in the absence of TH, co-repress the expression of many genes involved in metabolic processes (such as growth factors, catabolic enzymes and many more). The interaction of TH (mainly T3) with THR induces a conformational change that triggers transcriptional activation of these genes (Wu and Koenig, 2000). This cascade of events is the molecular link between dietary iodine uptake and metabolism, and the basis of cell growth, heart rate control, basal metabolic rate, thermogenesis, development and many other fundamental processes in vertebrates. In addition, it has been shown that TH have non-transcriptional effects (Davis et al., 2016) and that further de-iodination produces precursors of an important class of signalling compounds called thyronamines (TAM), which mainly act as neuro-modulators (Piehl et al., 2011). Low levels of TH induce the increased secretion of the peptidic thyroid stimulating hormone (TSH) from the pituitary gland. TSH tightly binds a G-protein coupled receptor (GPCR) located in the basolateral membrane of thyrocytes (TSH receptor, TSHR), which in turn upregulates the synthesis of thyroglobulin (TG) and other proteins needed for the synthesis of TH (Ortiga-Carvalho et al., 2016). Because of their central role in vertebrate biology, TH homeostasis is critical at all stages of life, and perturbations of the iodine cycle and hormonogenesis have severe consequences on human health. Among the factors that are known to directly affect TH synthesis are iodine intake through nutrition, genetic mutations, auto-immune diseases, environmental factors. Some relationship of dis-hormonogenesis with thyroid cancers have also being observed (Fiore et al., 2015). Over the last 50 years, many clinical and cellular studies have been conducted to uncover causal relationships between disease phenotypes and dis-hormonogenesis and to evaluate their impact on the population (Knezevic et al., 2020; Persani et al., 2018; Taylor et al., 2018). At the same time, developments in genomics, proteomics, imaging and three-dimensional tissue culture have provided powerful analysis tools and models to understand thyroid physiology (Antonica et al., 2017; Gillotay et al., 2020; Samimi et al., 2021; Wright et al., 2021). Nevertheless, still, many molecular mechanisms in thyroid biology, and the iodine cycle in particular, are yet to be elucidated. For example, what are the precise mechanisms determining the relative amounts of T4 and T3 produced by the thyroid gland and how is their ratio finely tuned? How do follicular structures balance TG production with TH release events? How is iodide access to the follicular lumen regulated? How is TG internalised in thyrocytes after hormone synthesis? What are the effects of common congenital mutations on TH synthesis and how can they be treated? How exactly do certain thyroid auto-immune diseases and cancers modify TH metabolism? How does the thyroid tissue structure change at a molecular level in goitres, the thyroid gland enlargements linked to many thyroid diseases? We would like to argue that learning more about the molecular architecture of thyroid proteins and their interactions will be required in most cases to make progress with these questions. So far, the structural characterisation of many proteins in the iodine cycle pathway has been an arduous task because of their complexity, heterogeneity, low abundance, frequent membrane location and biochemical instability (Citterio et al., 2019). Recent technological advances in electron cryo-microscopy (cryo-EM) and electron cryo-tomography (cryo-ET) promise to overcome at least some of these hurdles and to enable the analysis of the challenging thyroid macromolecular complexes, even at true atomic resolutions (Nakane et al., 2020). Hence, today, the application of modern structural biology offers a great opportunity to expand our mechanistic understanding of thyroid biology.

## 2 3D structures of proteins involved in thyroid hormone metabolism

**Fig. 2:**
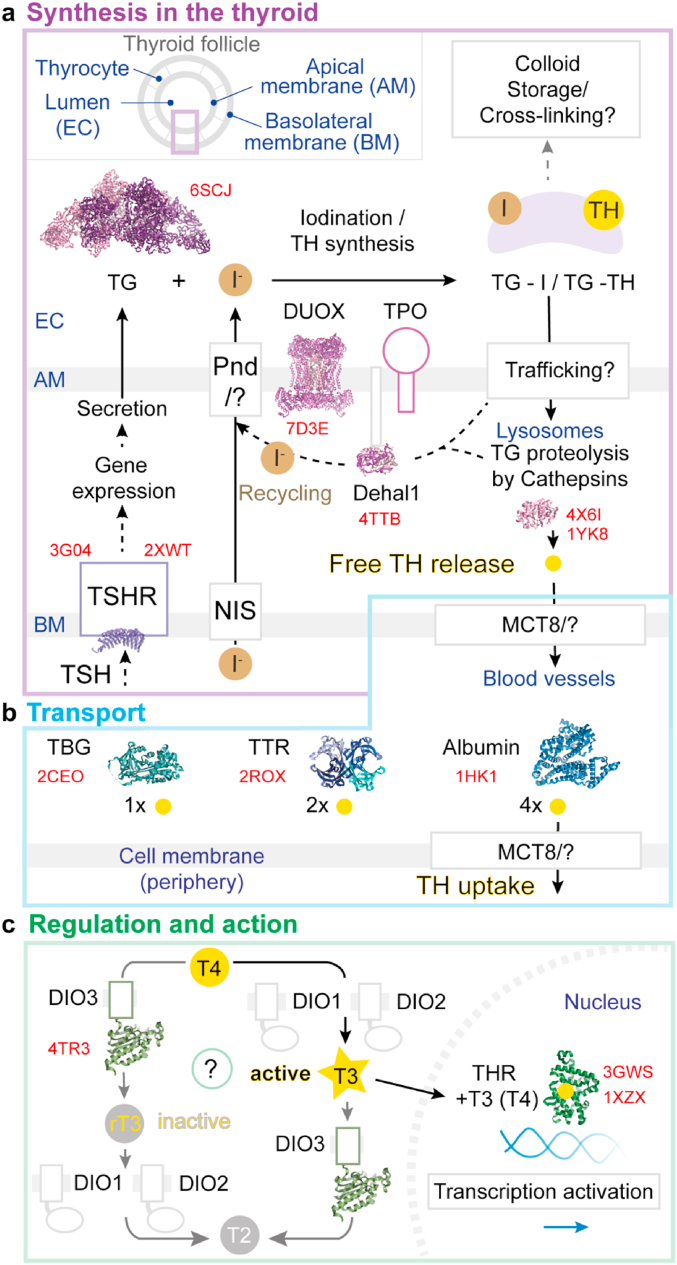
**Overview of the main proteins involved in the thyroid hormone pathway and their atomic structures.** Proteins involved in the synthesis (a), transport (b) and regulation/action (c) of thyroid hormones (TH). Protein Data Bank (PDB) identifiers are provided in red. White squares indicate unresolved protein moieties. Question marks indicate poorly understood mechanisms or structures. Inset in a): Schematic representation of a thyroid follicle. TSHR: TSH receptor, NIS: sodium/iodide symporter, Pnd: pendrin, TG: thyroglobulin, DUOX: dual oxidases, TPO: thyroperoxidase, Dehal1: iodo-tyrosine deiodinase, MCT8: monocarboxylate transporter 8, TBG: thyroxine-binding globulin, TTR: transthyretin, DIO: deiodinase, THR: thyroid hormone receptors.

In Fig. 2, we compiled an overview of the macromolecules involved in the three main stages of TH metabolism: TH synthesis (in the thyroid gland), TH transport (carriers in the blood and transport through membranes), TH effectors and their regulation (in peripheral tissues). Below we list and discuss the currently known atomic structures of the proteins that participate in these processes. In addition, we highlight the structures that remain to be determined to further advance our molecular understanding of the iodine cycle and thyroid biology.

### 2.1 TH synthesis in the thyroid

The thyroid gland follicles are monolayers of epithelial polarised thyroid cells, the thyrocytes, that enclose the lumen or extracellular compartment (EC). The membrane facing the EC is named apical membrane (AM), and the one facing the outside is the basolateral membrane (BM). The compositions of the AM and BM are very different, and this is crucial to allow TH synthesis in the EC. The outward-facing basolateral membrane (BM) contains TSH receptors (TSHR) that belong to the G-protein coupled receptors family of proteins (GPCR) (Parmentier et al., 1989). TSHR is also marginally expressed in other tissues (Williams, 2011). Upon binding to the stimulating TSH and other extracellular ligands, TSHR undergoes conformational changes in its membrane and cytosolic portions to trigger cAMP-mediated signalling cascades (e.g. through mitogen-activated protein kinase, MAPK) that directly act on gene expression. TSHR activation induces transcription, secretion and post-translational modifications, controlling thyroid cell growth, differentiation and TH synthesis (Kleinau et al., 2017). Importantly, TSHR is an antigen in autoimmune thyroid disorders (e.g. Graves' disease) (Kleinau and Vassart, 2000; Rapoport and McLachlan, 2007). Exploiting the binding to an autoantibody fragment Sanders and co-workers managed to obtain the first atomic structure of TSHR's extracellular domain (Sanders et al., 2007). This work has been an important step towards a molecular understanding of TSH stimulation by different ligands and helped to mechanistically link known disease mutations with their phenotypes (Kleinau and Vassart, 2000; Morshed and Davies, 2015; Núñez Miguel et al., 2016; Sanders et al., 2011). Despite extensive modelling efforts of the molecular signal transduction within TSHR's membrane-spanning domain, a full-length structure of TSHR would be required to aid the rational development of small-molecule inhibitors or activators (Davies and Latif, 2015; Grassi et al., 2021; Sun et al., 2019).

Among many other thyroid-specific genes, TSHR action controls the expression of another integral membrane protein in the outward-facing BM, the sodium iodide symporter (NIS), which responsible for iodine uptake into the thyroid, salivary and mammary glands (Dai et al., 1996; Portulano et al., 2014; Tazebay et al., 2000). Once in the cytoplasm, iodide is exported to the follicle lumen/EC mainly by pendrin, located in the EC-facing AM. This process is likely assisted by other transmembrane proteins, e.g., anoctamin-1 and the chloride channel CLC-5 (Cangul et al., 2018; Fong, 2011; Silveira and Kopp, 2015; van den Hove et al., 2006), the structures of which are currently unknown. Atomic models have been predicted from other homologous family proteins with significant sequence identity to understand their activities (Zhekova et al., 2020). However, experimental structures will be needed to help move us towards a complete understanding of the iodide specificity and the flux of iodide to the EC (Llorente-Esteban et al., 2020; Thompson et al., 2019).

TSHR also stimulates the secretion into the EC of a large dimeric glycoprotein, thyroglobulin (TG), that assembles into a cross-linked matrix within the EC, termed colloid (Berndorfer et al., 1996). TG has two principal functions: via iodination of its many solvent-exposed tyrosine amino acid moieties, TG serves as a reservoir of iodine and as the precursor of TH (Citterio et al., 2019). In the thyrocyte-enclosed EC, which functions as an extracellular reaction chamber, the apical membrane NADPH-dependent dual oxidase (DUOX) synthesises H_2_O_2_ (De Deken et al., 2000; Dupuy et al., 1999; Grasberger and Refetoff, 2006). H_2_O_2_ is then used by thyroperoxidase (TPO), a membrane-anchored heme-peroxidase, to convert iodide into oxidised and reactive iodine species (Kimura et al., 1989). These react with TG's accessible surface tyrosines (about 60 out of 132 total tyrosine residues) by generating mono- and di-iodo-tyrosine moieties (MIT, DIT). The reaction is thought not to involve direct contact between TG and TPO (Coscia et al., 2020). When two DIT or a DIT and a MIT are close in space and in flexible and solvent-exposed regions of the protein, they spontaneously undergo a side-chain rearrangement. This leads to the transfer of an iodo-substituted ring to the other, generating thyroid hormones within the TG's polypeptide chain. The recent cryo-EM structure of human thyroglobulin combined with *in vitro* hormonogenesis assays revealed that this reaction happens in precisely seven sites per TG dimer, generating as many TH molecules. Presumably, all the other iodinated tyrosines on TG that are not arranged in such pairs provide iodine storage (Coscia et al., 2020). TPO has been targeted by small molecules to inhibit TH synthesis (Furman, 2017). Apart from the catalytic peroxidase domain, TPO also contains CCP-like domains and an EGF-like domain that currently have no known functions within the TH synthesis context, but include the main region of antigenicity (McLachlan and Rapoport, 2007). As for many other membrane-bound components of the thyroid, the full-length structure of TPO is yet to be revealed. However, modelling and electron microscopy studies have provided insights into the general TPO three-dimensional architecture and its autoantibody binding modes (Le et al., 2015; Williams et al., 2018). It has been proposed that TPO and DUOX may physically interact in the AM to more efficiently produce TH (Fortunato et al., 2010). Very recently, the structures of both mouse and human DUOX enzyme have also been determined at atomic resolution by cryo-EM (Sun, 2020; Wu et al., 2021). While the human DUOX sample mainly adopts a tetrameric form (a dimer of dimers between DUOX1 and DUOXA1), in the mouse homologue both a heterodimeric and a tetrameric form of DUOX have been observed. It is still unclear what the physiological implications of the two oligomerisation states are, but further studies based on these structures promise to clarify this potential regulatory feature in DUOX. Notably, the structures from Wu and co-authors revealed significant conformational changes that promote enzymatic activation upon calcium binding, suggesting modulation of H_2_O_2_ production upon variations of intracellular calcium concentrations (Wu et al., 2021). Taken together, the DUOX structural insights lay the basis for more focused research on the role of this pivotal enzyme in thyroid hormonogenesis, on the impact of its mutations known to cause hypothyroidism and on the development of new drugs modulating its activity (Nie et al., 2015; Weber et al., 2013). Besides, the DUOX structures will be a valuable resource to investigate the putative TPO-DUOX interaction through mutagenesis and to probe their coupled enzymatic activity.

**Fig. 3:**
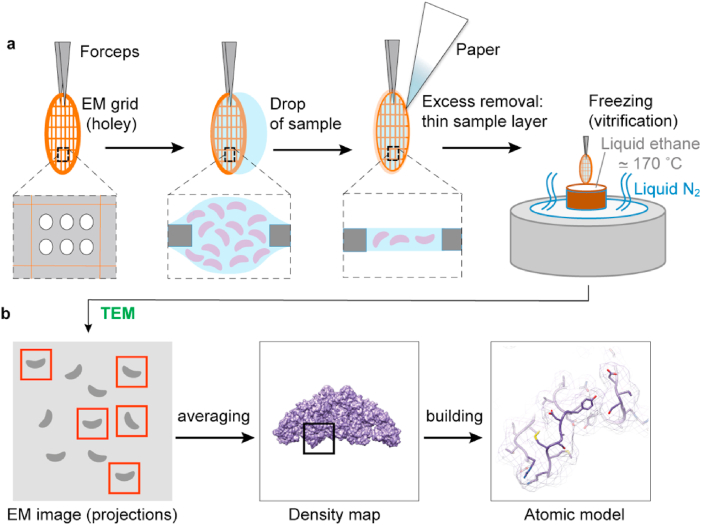
**Schematic of the single-particle cryo-EM methodology.** Simplified overview of single-particle cryo-EM sample preparation (a) and transmission electron microscopy (TEM) image analysis, resulting in an atomic model (b). a) EM support samples are metal mesh grids covered with a thin film containing tiny holes of about 0.6–2 μm. An approximately 3 μL drop is applied to the grid, and excess liquid is blotted away to obtain a very thin layer (about 30 nm thick) of solution in the holes. The grid is then vitrified in liquid ethane and inserted into the transmission electron microscope for imaging. b) The resulting micrographs are 2D projections of the 3D objects in the thin vitrified layer. Single objects within the image representing macromolecules are selected (red boxes) and averaged first in two dimensions and then combined to reconstruct a 3D density map of the imaged molecular solution. Finally, this map is used to build and refine the atomic model of the molecule.

Going further along the iodine cycle as depicted in Fig. 2, TG containing iodo-tyrosines (MIT, DIT) and TH (TG-I, TG-TH) is translocated from the EC into the thyrocytes by endocytosis. Although several import mechanisms and receptors have been suggested, the nature of TG endocytosis remains poorly understood (Botta et al., 2011; Lisi et al., 2003). Once back in the thyrocytes, TG is mainly directed to lysosomes, where it undergoes proteolytic cleavage, causing the release of TH from the polypeptide chain. The activity and expression levels of lysosomal proteases of the cathepsin family play a significant role in TH yields and their successive transport into the bloodstream (Jordans et al., 2009; Venugopalan et al., 2021). Cathepsins, and especially cathepsin K, are molecular targets for the regulation of thyroid function by small molecules, and their structures have hence been well studied in isolation and in the presence of inhibitors (Barrett et al., 2005; Borišek et al., 2015; Dauth et al., 2011; Gunčar et al., 1999) (Fig. 3a). Interestingly, it was shown that cathepsins are also secreted into the EC, potentially enabling the proteolytic pre-processing of TG (Brix et al., 2001). The iodine bound to non-hormonogenic iodo-tyrosines in TG is recycled by the iodo-tyrosine deiodinase Dehal1 (also called IYD). This enzyme does not de-iodinate TH, but only and specifically iodo-tyrosines. Dehal1 is anchored in the lumen/EC-facing AM of the thyrocytes, and its catalytic domain most likely resides in the thyrocytes' cytoplasm (Gnidehou et al., 2004). The crystal structure of the soluble domain of human Dehal1 allowed to decipher the role of the cofactor flavin mononucleotide in the reductive tyrosine de-iodination mechanism and to clarify differences with other iodothyronine deiodinases (Hu et al., 2015).

### 2.2 TH transport

Leaving the thyroid gland, TH liberated from TG by cathepsins are released into the bloodstream by membrane monocarboxylate transporters (MCT), mainly MCT8 (Friesema et al., 2003; Felmlee et al., 2020). The same system allows the transport of TH across membranes of cells other than thyrocytes. Furthermore, this transport may be facilitated by other amino acid carriers with a lower affinity for TH (Krause and Hinz, 2017). Mutations affecting MCT8 function and reduced levels of MCT8 dramatically affect thyroid and brain functions (Bernal et al., 2015). Knowledge of the detailed structures of the TH transporters could enable us to better understand the impact of the various known mutations on the transport reaction. Tremendous advances have been made recently with the structure determination of similar membrane solute carriers, MCT1 and MCT2, laying the basis for a detailed characterisation of MCT-TH transport (Lee et al., 2019; Wang et al., 2020; Zhang et al., 2020). Free TH are moderately hydrophobic and hence are delivered from the bloodstream to peripheral tissues by a system of three serum carrier proteins: thyroxine-binding globulin (TBG), transthyretin (TTR) and also albumin (Fig. 2b). The atomic structures of serum TH carriers and their TH binding stoichiometries and affinities have been elucidated (Refetoff, 2000). TBG, TTR and albumin can bind 1, 2 and 4 TH molecules and with decreasing binding affinities, respectively, with TBG being the main TH containing carrier in the blood. TTR is crucial for the delivery of T4 to the brain (Chanoine et al., 1992).

### 2.3 TH regulation and action

The thyroid gland mainly releases T4 into the bloodstream, and only small amounts of T3 (Rousset et al., 2000). The effect of TH on peripheral cells throughout the organism is principally transmitted through the conformational switch induced in TH receptors (THR) by T3, and to a lesser extent, by T4 (Sandler et al., 2004). THR binds DNA upstream of the majority of metabolic genes and does so in complex with co-repressor proteins to inhibit transcription in the absence of TH. To release repression, mostly T3, the active TH form, interacts with THR, and a resulting conformational change in THR exposes protein surfaces that allow transcription factors to be recruited and to start gene expression. As might be expected, given their wide-ranging roles in so many contexts and cell types, THR have a variety of isoforms that bind with varying affinities TH and are often tissue-specific. Extensive structural studies of THR have unravelled the molecular details of transcription activation upon binding to both T3 and T4 (Cheng et al., 2010). Mutations in THR modify the sensitivity to TH and can be mechanistically linked to TH hormone treatment resistance, which leads to detrimental effects in the affected individuals (Ortiga-Carvalho et al., 2014). The relative concentrations of T4 and T3 are regulated by a system of three iodothyronine-deiodinase enzymes (DIO) (Bianco and da Conceição, 2018; Bianco and Kim, 2006; Luongo et al., 2019). The important atomic structure of the soluble catalytic domain of DIO3 shows that this enzyme has a thioredoxin fold and that the deiodination reaction is exerted by a reductive mechanism involving a catalytic seleno-cysteine residue (Schweizer et al., 2014). Three different DIO enzymes de-iodinate different iodothyronine positions, activating T4 into T3 (DIO1/DIO2) or inactivating T4 into rT3 (DIO3) (Fig. 2c). DIO1 and DIO3 are anchored to the cell membrane, while DIO2 is anchored within the ER membranes. It has been shown that DIO2 is inactivated by ubiquitylation and subsequent proteasome degradation via the TEB4 and WSB-1 ubiquitin ligases. Strikingly, the latter have tissue-specific expression levels, with direct effects on local T3 concentrations (Curcio-Morelli et al., 2003; Dentice et al., 2005; Zavacki et al., 2009). It is important to point out that DIO enzymes are distinct from the iodo-tyrosine deiodinase Dehal1/IYD mentioned earlier, which recycles iodide in the thyroid gland (section 2.1). A complete understanding of T4 processing will also require knowledge of the atomic structures of selenium-containing DIO1 and DIO2. Further deiodination of T3 and rT3 leads to 3,3′-diiodo-L-thyronine (T2), which also stimulates THR and has metabolic effects (Senese et al., 2018). TH de-iodination coupled with other chemical modifications lead to the synthesis of thyronamines (TAM) and other TH derivatives. The biosynthetic pathways of this class of novel thyroid hormone derivatives, mainly affecting brain function (Zucchi et al., 2019), remain to be further elucidated at the genetic, biochemical and structural levels and provide an interesting area of research.

## 3 Recent advances in cryo-EM

Until recently, atomic structure determination of macromolecular complexes was dominated by X-ray crystallography. This method is based on the diffraction of X-rays by a crystal of the sample, i.e. a three-dimensional (3D) array of molecules in the solid state. The diffraction pattern intensities, combined with phases derived experimentally or from a homologous structure, allow obtaining a 3D electron density map of the molecule of interest. This map is then used to build and refine an atomic model that agrees best with the observed map. With many recent improvements of X-ray sources, data processing workflows and automation, crystallographic structures can be obtained in minutes as long as suitable crystals are available. Nowadays, the method is ideally suited for small proteins and complexes (<100 kDa) and also high-throughput small molecule and fragment screening. Recently, for example, the binding of new inhibitors of the thyroid onco-target B-RAF was visualised at atomic resolution by X-ray crystallography (Cotto-Rios et al., 2020). The main (and serious) bottleneck of the technique is the need to obtain ordered and well-diffracting crystals, which might require large amounts of sample. This can be very challenging for heterogeneous samples, multi-domain proteins, glycosylated and membrane proteins. Truncation of non-conserved regions or even domains or proteolysis is widely used to remove flexible areas or select a stable conserved core that can be crystallised (e.g. removal of membrane portion in TSHR and DIO3). A complementary technique, Nuclear Magnetic Resonance (NMR), produces ensembles of atomic coordinates of a sample in solution and depends on the analysis of the magnetic perturbations of the nuclei in the protein sample (for example of hydrogen, carbon and nitrogen) in the presence of a powerful magnetic field. The method is straight forward for small molecules, oligonucleotides, peptides, and proteins soluble at high concentration. Complete structural determination by NMR becomes progressively more difficult as the size of the macromolecule increases and is basically impossible over the 30 kDa limit. Still, this technique can provide detailed information on particular residues and atoms in large complexes treated with specific isotopic labelling. Notably, NMR is the only method that can reveal the structure and dynamics of intrinsically disordered peptides and proteins (Snyder, 2005; Yee, 2005).

The third method, transmission electron microscopy (TEM), allows obtaining highly magnified images (in the range of 100 k x magnification) produced by the scattering of high-energy electrons by a very thin specimen (from 10 nm to a few hundred nm). In order to allow electrons to travel through the instrument, the imaging must be done in a high vacuum and cannot be normally performed directly on solutions, which would evaporate. Originally, TEM played an important role in cellular structural biology by providing morphological details of tissues embedded in plastic resin and stained with heavy atom solutions. These samples resist to evaporation and can be imaged after “fixing” in a transmission electron microscope at room temperature. Similarly, by staining large macromolecular complexes with heavy metal atom salt solutions, it is possible to visualise their overall shape at around 30 Å resolution. This technique, called negative staining EM, was used to image thyroglobulin for the first time (Berg et al., 1980). In the 1980s–90s, a new sample preparation method was developed, in which a thin layer of solution is very rapidly cooled to liquid ethane temperature (approximately at −170 °C), a process known as vitrification. This procedure (Fig. 3) prevents the formation of ice crystals that would strongly scatter the electrons and allows the imaging by TEM of protein samples in a close-to-native hydrated state. The TEM analysis of vitrified samples is now named electron cryo-microscopy, or cryo-EM. Because proteins are made of light atoms similar to the surrounding solution, cryo-EM images of biological samples are characterised by very poor contrast. In addition, images are very noisy because electrons damage biological molecules very rapidly, and only low electron fluxes can be used. High-resolution well-defined images of the sample can however be obtained by computational averaging of many selected images of the individual molecules in a micrograph (often hundreds of thousands). Combining these images corresponding to multiple orientations of the sample in solution (Fig. 3b), it is possible to calculate a 3D density map sufficiently detailed to build within it an atomic model, similar as for X-ray crystallography. However, the maps obtained with the two techniques reveal different entities (electron density in the case of X-ray, Coulomb potential in cryo-EM). Initially, the method produced near-atomic structures (3–4 Å) for large and symmetrical assemblies only (typically viruses). In the last decade, enormous efforts have been put into improving cryo-EM electron sources, sample supports, electron detectors and image processing methods to increase the signal-to-noise ratio of cryo-EM images and the resulting maps. These recent technical advancements led to the so-called “resolution revolution” (Kühlbrandt, 2014), which earned cryo-EM pioneers Jacques Dubochet, Joachim Frank and Richard Henderson the Nobel Prize in Chemistry in 2017 (Frank, 2017). Nowadays, a few microliters of a moderately concentrated protein solution are often sufficient to obtain near-atomic structures of proteins and complexes bigger than approximately 70 kDa at resolutions of 2–3.5 Å (Khoshouei et al., 2017; Nakane et al., 2020). A key feature and advantage of single-particle cryo-EM analysis is the possibility to resolve many different conformations adopted in solution by the macromolecules, obtaining a dynamic view of molecular states in solution (Nakane et al., 2018). Moreover, following in the steps of X-ray crystallography before it, fully automated procedures in cryo-EM are being developed (Zivanov et al., 2018), making the method faster and more accessible. In parallel with single-particle cryo-EM, major improvements have also been reported in the sample preparation of thin cellular specimens (<300 nm thick, the approximate limit for high-energy electron transparency) (Schaffer et al., 2019). For unique non-redundant objects (e.g., cell sections) that cannot be averaged, single TEM images cannot yield a 3D map of the sample. However, it is possible to collect a series of TEM images at different angles while tilting the sample to provide different views. The resulting so-called “tilt series” of two-dimensional images can be computationally aligned and transformed to produce a 3D volume of the object of interest (electron cryo-tomography or cryo-ET). For mechanical and geometric reasons, it is currently impractical to perform the necessary full 180 °C rotation of the specimen. This leads to 3D reconstruction artefacts due to the lack of information in some directions (the “missing wedge” problem). Besides, in cryo-ET, due to the stage movement during multiple low-dose electron exposures, it is challenging to obtain atomic resolution reconstructions, even for repeated large objects within the sample (e.g. ribosomes in ER and viruses). Cryo-ET of fully asymmetric samples is currently limited to about 30 Å resolution, not enough to recognise even large proteins in a cellular context, but hopefully, further advances in detector efficiency, microscope technology, phase plates and image processing procedures will lead to cryo-ET tomograms that achieve better than 10 Å, to recognise secondary structure elements (Hutchings and Zanetti, 2018; Schorb et al., 2019).

So far, the majority of structures in the iodine cycle (Fig. 2) have been determined by X-ray crystallography. However, going forward and as explained earlier, a large proportion of still unknown mechanisms involves integral or membrane-binding proteins, large extracellular complexes or rare and difficult enzymes. These may be hard to crystallise for their compositional and conformational heterogeneity or low expression yields (for example, the full-length TSHR and TG-I). Cryo-EM now provides an excellent opportunity to try or retry to investigate the architecture of these arduous targets. Below we describe as a case study how single-particle cryo-EM enabled the large and complex structure of human thyroglobulin to be resolved (Coscia et al., 2020) and how this method could potentially help the study of other challenging thyroid proteins.

## 4 Thyroglobulin cryo-EM structure determination: a case study

Despite its pivotal role in thyroid hormonogenesis and iodine storage, TG's structural characterisation was previously limited to low-resolution negative staining EM (Berg et al., 1980). TG is a secreted dimer of about 600 kDa, with extensive glycosylation. Deglycosylated samples were used for many years for crystallisation trials of TG from different vertebrate sources, but the resulting crystals were never ordered enough for structure determination. TG contains many cysteine-rich small domains (thyroglobulin repeats) and a larger acetylcholine esterase like (Ache) domain at the C-terminus (Vassart et al., 1985). From limited proteolysis or through sequence analysis, it was also not possible to identify the boundaries of conserved structural units that then could become amenable to crystallographic analysis. Therefore, the successful route was to abandon crystallography and to determine the full-length TG structure taking advantage of the recent developments in cryo-EM. When initially trying to vitrify TG from bovine, porcine and mouse sources, aggregation and degradation were observed, with varying severity. Improved biochemical behaviour was observed with commercial human TG extracted from goitre (of undisclosed pathology). Mild deglycosylation and further purification resulted in a very diluted sample at less than 0.05 g/L that was not amenable to standard cryo-EM. Therefore, the sample was applied to EM grids covered by a graphene oxide layer. Because TG adheres to this support, its density on the grid is enhanced. Using this sample preparation and the (then) latest TEM microscopes and direct electron cameras (FEI Titan Krios operated at 300 kV and Gatan K2 camera), the first native snapshots of TG were obtained. An initial reconstruction yielded a map that was only defined around the central Ache domain but was very blurred in the lateral portions of the dimer. Collecting a large dataset and performing *in silico* selection of the most compact particles was essential to obtain a final map at near-atomic 3.5 Å resolution. This level of detail was necessary to resolve and discriminate among the 17 related cysteine-rich TG repeat domains and identify the structural regions, which were called NTD (N-terminal domain), core, flap, arm and CTD (C-terminal domain) (Coscia et al., 2020). TG is a highly intertwined dimer in which all domains interact with the central Ache-like core. The NTD was confirmed to be an autonomous hormone-forming domain that is loosely bound to the main body of the protein (Veenboer and de Vijlder, 1993). As mentioned earlier, the TG structure allowed to map all of the key tyrosine pairs and to identify the exact number of TH generated by a TG dimer. The iodo-tyrosine pairing giving rise to T4 (and likely T3, although not detected using *in vitro r*eactions with recombinant TG), could be reproduced effectively in a much less complex scaffold protein, the bacterial maltose-binding protein (MBP). It remains to be established if the highly conserved TG multi-domain architecture has other functional implications than hormone biosynthesis. Moreover, the number of hormonogenic sites is variable across species, and the relationship between early and late sites is an interesting aspect that the structure will permit to investigate in the future (Lamas et al., 1989). In this context, it is worth mentioning that TG seems to be able to form different amounts of T4 and T3 *de novo*, depending on post-translational modifications and other factors which are yet to be elucidated (Citterio et al., 2018). In the final TG cryo-EM map, even after deglycosylation, many glycans were still present and visible, and some interacted with surface residues, contributing to the overall stability of TG. Notably, at position Asn2013, the glycan chain is sandwiched between two subunits. This is likely to contribute to the stability of the dimer, as it was also highlighted by proteomic studies (Wright et al., 2021). It is therefore also unsurprising that TG expressed in other systems than mammalian (or probably other vertebrate) cells produced aberrant TG oligomers and were not suitable for structural analysis. This finding highlights that glycan-protein interfaces are potential sites for detrimental mutations that affect TG assembly and therefore TH synthesis. It was proposed that TG can autoregulate its own expression and hormone release (Sellitti and Suzuki, 2014; Sue et al., 2012; Suzuki et al., 2011) by an unknown molecular mechanism. Moreover, its extracellular and lysosomal proteolytic cleavage is pivotal to determine the concentration of release of TH, but this complex process remains poorly understood. It must be noted that 7% of TG's sequence was not resolved in the cryo-EM map, and these regions might be important for these functions and interactions with its partners during secretion, internalisation and lysosomal processing. All these mechanisms might well involve conformational deviations from the structure of un-iodinated TG, which may now be investigated by cryo-EM. Finally, the architecture of TG provides a framework to study congenital mutations and the role of TG in autoimmune diseases (Latrofa et al., 2013). The cryo-EM structural determination of TG also highlights and confirms several biochemical features and prompts many questions that can be addressed by rational design of mutants *in vitro* and *in vivo* or in organoid models (Antonica et al, 2012, 2017). Similarly, glycosylated and dynamic (especially membrane-bound) proteins in the thyroid biosynthetic pathway can now be discovered by cryo-EM analysis. A good example is represented by the recent DUOX cryo-EM structures that could not be previously determined by X-ray crystallography (Sun, 2020; Wu et al., 2021).

## 5 Towards a complete mechanistic understanding of thyroid biology

Thyroid hormones are essential iodine-containing small molecules that control a large number of fundamental processes in vertebrates. Thyroid function and pathogenesis have been studied for many years from the clinical, genetic, cellular and biochemical perspectives, elucidating essential aspects of thyroid hormone metabolism in health and disease. Furthermore, structural biology, mainly X-ray crystallography, providing atomic snapshots of some of these processes, has greatly contributed to our understanding of their mechanisms and to interpret and understand the effects of congenital disease mutations. For example, we have learned about THR's conformational changes upon binding to different thyroid hormones, the mechanism of stimulation of TSHR by autoantibodies and the deiodination of T4 to rT3 by DIO3. Nevertheless, many aspects of the homeostasis of the TH ratio and the relationship with follicular phenotypes currently remain obscure and will significantly benefit from further structural investigations (Rurale et al., 2020). For example, goitre is a typical phenotype that may have a multitude of underlying reasons: iodine deficiency, autoimmune diseases or mutations in the biosynthetic pathways (Can and Rehman, 2020). What are the precise molecular determinants and their contribution in driving the macroscopic changes of the follicular tissue? A detailed structural and mechanistic description of the follicle cycle, from TG secretion to TH release, under different conditions will be critical to provide these insights and to suggest ways to tune/adjust the phenotype *in vivo*. The revolution in EM capabilities, especially cryo-EM and cryo-ET, move us closer to study these underexplored dynamic processes involving glycosylated, membrane-bound, heterogeneous, or rare complexes, which have been precluded from detailed studies in the past (Coscia et al., 2020). Eventually, cryo-EM could be performed directly on proteins extracted from natural or engineered tissues or even *in situ* (Hoffmann and Giandomenico et al., 2020; O'Reilly et al., 2020). In this vision, cryo-EM will be synergistically integrated with data from single-cell transcriptomics, proteomics and *in vivo* cell imaging to provide a multidimensional, predictive and more robust model of the entire thyroid system.

## Declaration of competing interest

The authors have no competing interest to declare.
